# Irreversible Electroporation to Treat Malignant Tumor Recurrences Within the Pelvic Cavity: A Case Series

**DOI:** 10.1007/s00270-017-1657-6

**Published:** 2017-05-03

**Authors:** L. G. P. H. Vroomen, H. J. Scheffer, M. C. A. M. Melenhorst, N. van Grieken, M. P. van den Tol, M. R. Meijerink

**Affiliations:** 1 Department of Radiology and Nuclear Medicine, VU University Medical Center, de Boelelaan 1117, 1081 HV Amsterdam, The Netherlands; 2 Department of Pathology, VU University Medical Center, de Boelelaan 1117, 1081 HV Amsterdam, The Netherlands; 3 Department of Surgical Oncology, VU University Medical Center, de Boelelaan 1117, 1081 HV Amsterdam, The Netherlands

**Keywords:** Ablation, Feasibility, Locoregional neoplasm recurrence, Pelvic region, Peripheral nerves

## Abstract

**Objective:**

To describe the initial experience with irreversible electroporation (IRE) to treat pelvic tumor recurrences.

**Methods:**

A retrospective single-center analysis was performed. Adverse events were recorded using Common Terminology Criteria of Adverse Events (CTCAE) 4.0. Clinical outcome was determined using pain- and general- symptom assessment, including Seddon’s peripheral nerve injury (PNI) types. Radiological outcome was evaluated by comparing baseline with three-month 18F-FDG PET-CT follow-up.

**Results:**

Eight patients (nine tumors [recurrences of primary rectal (*n* = 4), anal (*n* = 1), sigmoid (*n* = 1), cervical (*n* = 1), and renal cell carcinoma (*n* = 1)]) underwent percutaneous IRE as salvage therapy. Median longest tumor diameter was 3.7 cm (range 1.2–7.0). One CTCAE grade III adverse event (hemorrhage) and eight CTCAE grade II complications occurred in 6/8 patients: vagino-tumoral fistula (*n* = 1), lower limb motor loss (*n* = 3; PNI type II) with partial recovery in one patient, hypotonic bladder (*n* = 2; PNI types I and II) with complete recovery in one patient, and upper limb motor loss (*n* = 2; PNI type II) with partial recovery in both patients. No residual tumor tissue was observed at 3-month follow-up. After a median follow-up of 12 months, local progression was observed in 5/9 lesions (4/5 were >3 cm pre-IRE); one lesion was successfully retreated. Debilitating preprocedural pain (*n* = 3) remained unchanged (*n* = 1) or improved (*n* = 2).

**Conclusion:**

IRE may represent a suitable technique to treat pelvic tumor recurrences, although permanent neural function loss can occur. Complete ablation seems realistic for smaller lesions; for larger lesions symptom control should be the focus.

## Introduction

Present local tumor recurrences are a challenge for any malignancy in any location. Tumors that recur in the pelvis may be even more challenging to treat. Malignancies that are notorious for their recurrence within the pelvis following radiotherapy and/or surgery are both female and male urogenital tract tumors and locoregional recurrences from gastrointestinal origin such as anorectal carcinomas [1, 2]. Due to ingrowth in or compression on peripheral nerves, these relapsing malignancies can cause aggravating pain and neural function loss. Many of these patients have undergone multiple prior resections resulting in scar and adhesions, most have undergone some form of systemic therapy, and many have received external beam radiation. The presence of extensive adhesions induced by previous surgical procedures and the risk of radiation-induced toxicity in a previously irradiated area precludes radical local treatment options such as repeat surgery [3] and stereotactic ablative body radiation therapy (SABR) [4, 5]. The risk of severe treatment-induced morbidity does not seem to outweigh clinical benefit [2, 6]. In general, therapy for this specific patient population primarily aims at prolonging the—preferably quality preserved—life span, and most patients will be referred to medical oncologists for either palliative chemotherapy or the best supportive care [7, 8].

Yet, selected patients can be offered other local treatment modalities such as radiofrequency ablation (RFA) or cryotherapy [9]. One important drawback of these thermal treatment modalities is the high risk of inducing thermal damage to important neural structures like the sciatic nerve or perisacral plexus, as well as to the intestines, bladder, ureters, and large pelvic vessels [10].

Irreversible electroporation (IRE) is an emerging ablation technique that is based on the application of an electric field across cells that alters the transmembrane potential. On reaching a sufficiently high voltage, the phospholipid bilayer structure of the cell membrane is permanently disrupted, inducing apoptosis [11, 12]. Electroporation is regarded to leave supporting tissue largely unaffected, so the structural integrity of large blood vessels and intestines is relatively preserved [11, 13]. Moreover, initially damaged axons may regenerate with complete recovery of function, according to preclinical animal studies [14–17]. For these reasons, IRE may prove a safe and feasible treatment option for patients with malignant tumor recurrences within the pelvis that are considered unsuitable for established focal therapies [14, 15, 17].

This retrospective study describes the preliminary single-center experience of eight patients (nine lesions) with locoregional malignant tumor recurrences within the pelvis that were treated with percutaneous IRE in terms of morbidity and disease control.

## Materials and Methods

A retrospective analysis was performed of all patients treated with IRE for malignant pelvic tumor recurrences that were considered unsuitable for additional resection, re-radiation, or thermal ablation techniques due to the vicinity of major nerves, prostate, ureter, or intestines. Further systemic chemotherapy was considered unfavorable. All patients were discussed in our weekly multidisciplinary oncology board, which consisted of at least a medical oncologist, radiation therapist, surgical oncologist, diagnostic abdominal and interventional radiologist, and pathologist. IRE was only performed if the oncology board unanimously agreed on the indication. Treatment with IRE was considered off-label use within the scope of an individual treatment decision. Patients were considered appropriate IRE candidates if they met the following criteria: (1) IRE was used as salvage therapy, (2) a reasonable functional reserve [American Society of Anesthesiologists (ASA) performance status ≤3], and (3) the absence of ventricular arrhythmias. Histopathological proof of recurring malignancy was required, and the lesions had to be proven 18F-fluorodeoxyglucose positron emission tomography (18F-FDG PET) avid prior to IRE. A history of epilepsy was considered an exclusion criteria. Local review board approval for the study was obtained, and all patients provided written informed consent. The study was conducted conformal to the guidelines for Good Clinical Practice and the Declaration of Helsinki.

### IRE Procedure

All procedures were performed under general anesthesia with propofol, sufentanil, and rocuronium and maintained with propofol and remifentanil. To define the three-dimensional measurements of the tumor and its vicinity to vital structures, a contrast-enhanced computed tomography (ce-CT) scan was performed, using multiplanar image reconstruction. The size and shape of the tumor, including a 5-mm tumor-free margin, determined the number and configuration of the needle electrodes. Needle electrodes with an exposure length of 15 mm were percutaneously positioned in and around the tumor under CT fluoroscopy guidance, aiming at an interneedle distance of 15–20 mm. The NanoKnife (AngioDynamics Inc, Latham, NY) was used to perform the procedure. A total of 100 pulses of 1500 V/cm with a 90-μs pulse length were delivered for each electrode pair. For larger tumors, the electrodes were pulled back 1.0 cm to ablate the superficial part of the tumor.

### Safety Assessment

Immediately after the procedure, a second ce-CT scan was acquired to assess the ablation zone and to detect early complications such as perilesional bleeding. All direct and indirect procedure-related complications were scored according to the Common Terminology Criteria of Adverse Events (CTCAE), version 4.0. Pain assessment was determined using the Visual Analog Scale (VAS) scores prior to IRE, and 24 h and 3 months after the procedure. Physical examinations were performed pre- as well as post-procedurally in order to identify IRE-induced nerve loss. Neurological impairment was evaluated using Seddon’s classification [18]. Based on the severity of the peripheral nerve injury (PNI), the prognosis, and the recovery time, three types of neural damage—neurapraxia, axonotmesis, and neurotmesis—are defined within the Seddon’s classification [18]. Neurapraxia describes the mildest type and refers to a block to conduction of nerve impulses but without interruption of the axon or perineurium [19]. Axonotmesis is the second type and refers to an injured axon, yet the surrounding connective tissue remains intact [19]. Neurotmesis is considered the most severe injury type in which the axon as well as the connective framework is damaged [19] (Table 1).

**Table 1:** Seddon’s classification

	Score	Tissue injured	Clinical findings	Prognosis
Neurapraxia	I	Myelin	Profound motor loss, paralysis lasting days–months Normal to minimal sensory involvement	Excellent
Axonotmesis	II	Myelin, axon	Complete motor loss with sensory involvement OR Complete motor loss with normal sensation	Fair
Neurotmesis	III	Connective sheath damage ranges from partial disruption of the endoneurium to complete disruption of the involved nerve	Complete motor loss Complete sensation loss	Poor

### Oncological Outcome

Outcome was evaluated comparing baseline with three-month 18F-FDG PET-CT, using both the revised Response Evaluation Criteria In Solid Tumors (RECIST) [20] and the PET Response Criteria In Solid Tumors (PERCIST) [21] to obtain primary and assisted efficacy rates, time to local progression (TLP), and time to distant progression (TDP). Imaging using PET-CT was chosen because of its ability to detect tumor residue or progression in an area that is characterized by a disturbed anatomy. Since IRE induces local inflammation post-IRE, three-month follow-up was preferred above monitoring at an earlier time point. Based on imaging features, the parameters for local site recurrence (LSR) were (a) tumor lesion increase of at least 20% in longest diameter compared to the baseline scan at 3 months post-IRE, or (b) a more than 30% increase in standardized uptake value corrected for lead body mass (SUL) peak or growth in lesion total lesion glycolysis by more than 75%, without signs indicative for inflammation or abscess formation. Primary efficacy rate was defined as the percentage of lesions without signs for LSR at least 3 months after the initial IRE procedure, according to the above-mentioned criteria. Assisted efficacy rate was defined as the percentage of tumors completely eradicated at least 3 months after the last procedure, including tumors that underwent repeat ablation(s) [22].

## Results

Between December 2012 and April 2016, eight patients (nine tumors) underwent percutaneous CT-guided IRE to treat recurrences of primary rectal (*n* = 4), anal (*n* = 1), sigmoid (*n* = 1), cervical (*n* = 1), and renal cell carcinoma (*n* = 1). Needle insertion and pulse delivery were successful in all procedures. Median longest tumor diameter was 3.7 cm (range 1.2–7.0). Patient and tumor characteristics are displayed in Table 2.

**Table 2:** Patient and tumor characteristics

Pt.	Sex	Age (years)	Primary tumor	Histopathology of primary tumor	Treatment of primary tumor	Number of lesions	Tumor size (mm) (width, depth, length)	Vulnerable structures at risk in close proximity of the tumor	Treatment(s) prior to IRE
1	Male	70	Rectal	Adenocarcinoma	Transanal endoscopic microsurgical (TEM) rectum resection	1	60 × 44 × 48 15 × 16 × 26^a^	Bladder wall, ureter, lumbosacral nerve plexus	Neo-adjuvant chemoradiation
2	Female	57	Anal	Squamous cell carcinoma	Chemoradiation + perineal resection	1	50 × 49 × 40	Sciatic nerve	Radiotherapy
3	Female	63	Cervical	Unknown	Chemoradiation	1	70 × 38 × 40	Sciatic nerve	Radiotherapy
4	Male	74	Rectal	Adenocarcinoma	Radiotherapy + perineal resection	1	27 × 29 × 29	Ureter, sciatic nerve	Neo-adjuvant chemoradiation and additional SBRT
5	Male	58	Rectal	Adenocarcinoma	Resection	1	36 × 24 × 51	Sacral plexus (in particular, S3 and S4)	Neo-adjuvant chemoradiation and additional SBRT
6	Male	48	Rectal	Adenocarcinoma	Chemoradiation + rectum resection	2	17 × 10 × 12 14 × 17 × 15	Prostate, ureter, pelvic splanchnic plexus	Neo-adjuvant chemoradiation
7	Female	66	Sigmoid	Adenocarcinoma	Resection	1	10 × 11 × 12	Ureter	Re-resection (2×)
8	Male	52	Renal cell carcinoma	Chromophobe carcinoma	Nephrectomy	1	21 × 20 × 30	Intestines	Resection and RFA

^a^Patient developed a marginal recurrence which was successfully retreated with percutaneous IRE (see text)

### Complications

There were no deaths within 90 days post-IRE. One patient experienced a delayed hemorrhage after restarting anticoagulation therapy 3 days after the procedure (CTCAE grade III). Eight CTCAE grade II complications occurred in 6/8 patients. Three patients showed lower limb motor loss [all PNI type II (axonotmesis)], with partial recovery in one patient. Two patients developed a hypotonic bladder [PNI type I (neurapraxia), and PNI type II (axonotmesis)] with complete recovery in one patient. Two patients showed upper limb motor loss, with partial recovery in both patients [PNI type II (axonotmesis)]. One patient developed a vagino-tumoral fistula following the IRE procedure. Procedure-related details including complications are summarized in Table 3.

**Table 3:** Procedure details, clinical and radiological outcome

Pt.	# Probes	# Pullbacks	Complications (CTCAE grade)	Complication characteristics	Seddon’s classification	Affected nerve(s)	Recovery neural function	Follow-up (months)	Time to progression (months)	
									TLP	TDP
1	5	1	–	–	–	–	–	36^†^	4/–*	3
2	6	2	II	Lower limb motor loss + sensory involvement Vagino-tumoral fistula	Axonotmesis	Sciatic nerve	Partial	11^†^	5	–
3	6	2	II II	Hypotonic bladder Lower limb motor loss + sensory involvement	Neurapraxia Axonotmesis	Pudendal plexus S2–S4 Sciatic nerve	Completely None	21^†^	5	5
4	4	1	II III	Slight deterioration of preexisting lower limb motor loss + sensory involvement Hemorrhage	Axonotmesis	Sciatic nerve	None	12^†^	6	6
5	6	1	II	Hypotonic bladder	Axonotmesis	Pudendal plexus S2–S4	None	17	7	–
6	4 4	1 0	–	–	–	–	–	9	–	9
7	3	0	II	Upper limb motor loss + sensory involvement	Axonotmesis	Femoral nerve	Partial	9	–	–
8	6	0	II	Upper limb motor loss + sensory involvement	Axonotmesis	Femoral nerve	Partial	4	–	–

*TLP* time to local progression, *TDP* time to distant progression

* Patient developed a marginal recurrence which was successfully retreated with percutaneous IRE (see text)

^†^Deceased

### Follow-up

**Fig. 1:**
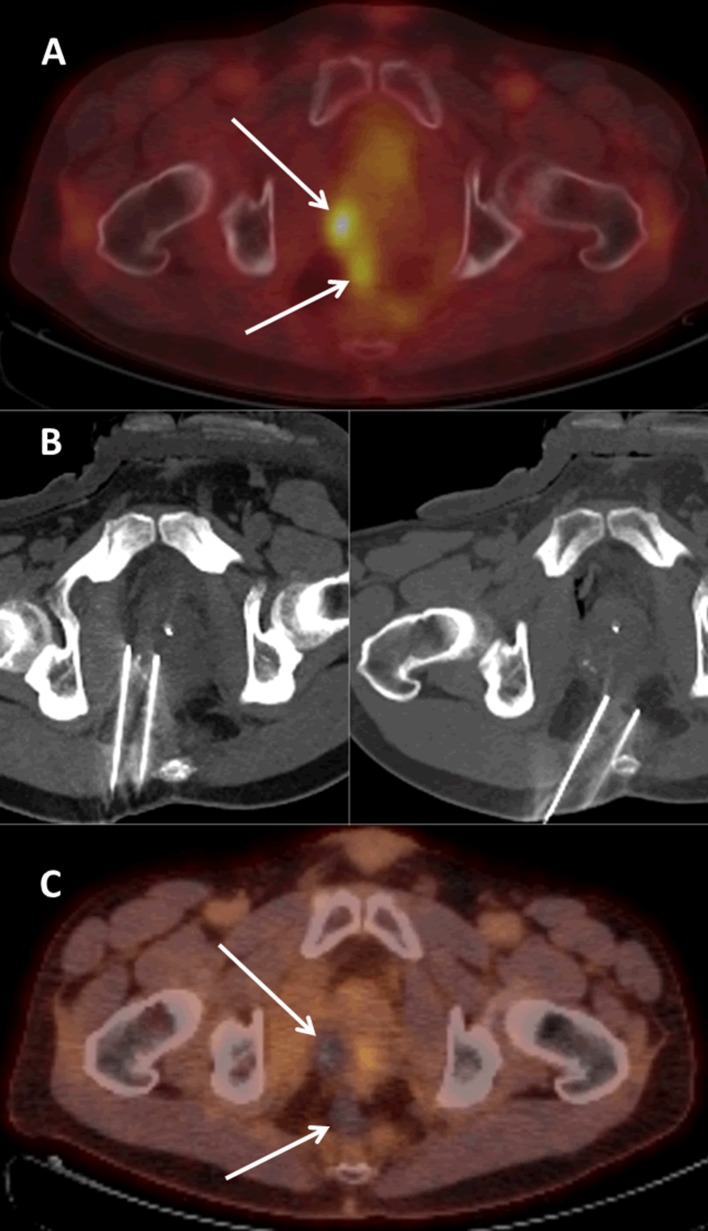
18F-FDG PET-CT image of a 48-year-old male patient with two small pathologically proven locoregional recurrences (*arrows*) of primary rectal adenocarcinoma in the precoccygeal and right peri-prostatic area (**A**). Nonenhanced CT scan showing the inserted needle electrodes prior to pulse delivery (**B**). 18F-FDG PET-CT image 3 months after IRE showing no signs for residual or recurring disease (**C**)

**Fig. 2:**
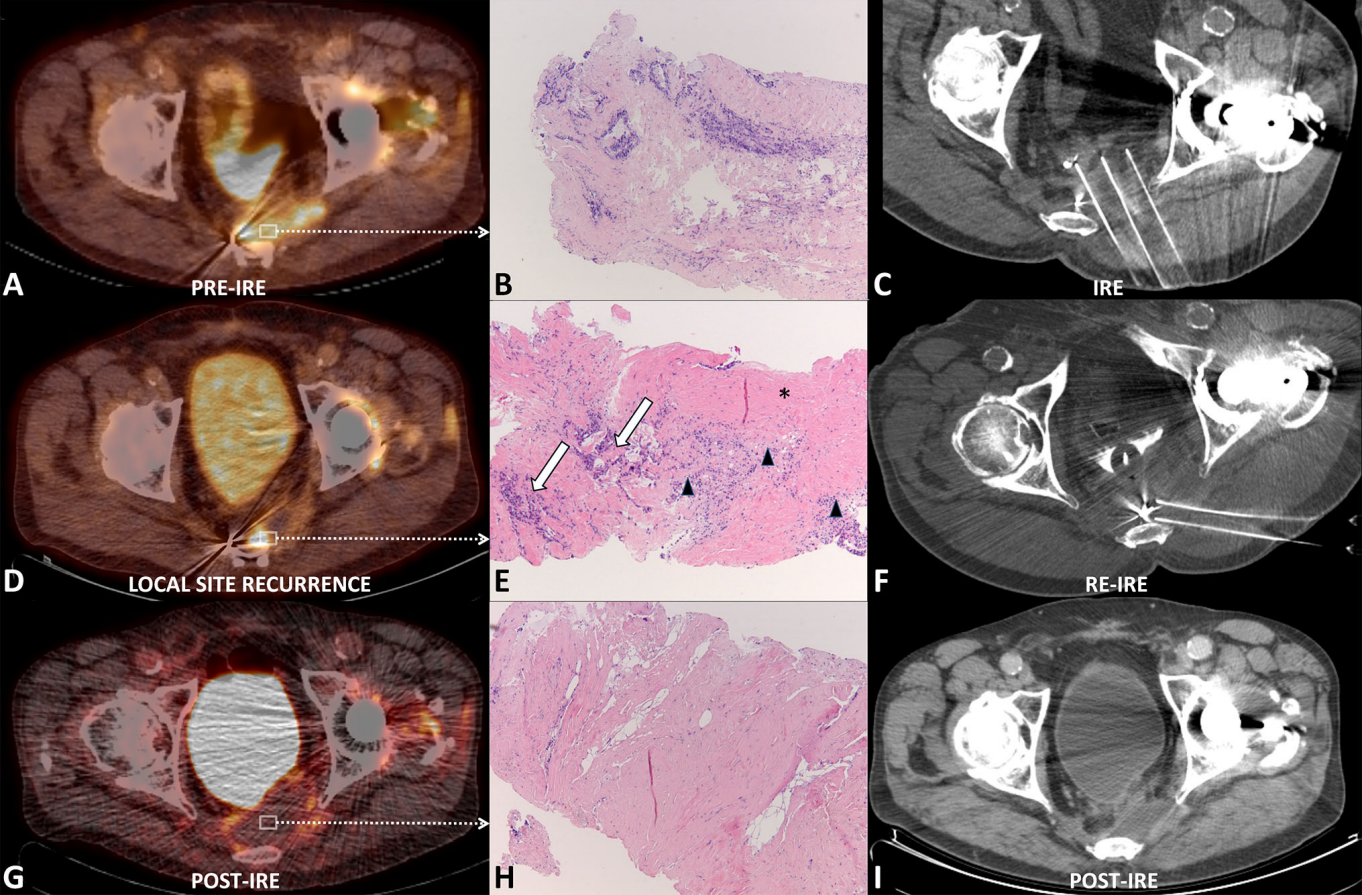
18F-FDG PET-CT image (**A**) of a 70-year-old male patient with an 18F-FDG avid 60-mm pathologically proven locoregional recurrence (*arrows*) of primary rectal adenocarcinoma in the left parasacral area. Pre-IRE biopsy (**B**) of the initial LSR showing malignant cells on hematoxylin and eosin (HE) staining. Nonenhanced CT scan (**C**) showing three of the inserted needle electrodes prior to pulse delivery during the initial IRE procedure. 18F-FDG PET-CT image (**D**) 4 months after the initial IRE procedure showing a LSR. Pre-IRE biopsy (**E**) of the LSR prior to the second IRE procedure showing malignant cells (*white arrows*) encompassed by inflammatory cells (*arrow heads*); both embedded in fibrotic tissue (*asterisks*) on HE staining. Nonenhanced CT scan (**F**) showing two of the inserted needle electrodes just before pulse delivery during the second IRE procedure. 18F-FDG PET-CT image (**G**) 3 months after the second IRE procedure showing no signs for residual or recurring disease. Post-IRE biopsy (**H**) of the ablated area after the second IRE procedure showing fibrotic tissue on HE staining. Nonenhanced CT scan (**I**) 6 months after the second IRE showing no signs of LSR

In all patients, no residual tumor tissue was observed at 3-month follow-up imaging. After a median follow-up of 12 months (range 4–36), four patients were still alive and four had deceased, respectively, 11, 12, 21, and 36 months after IRE. Although no LSR’s have been objectified according to conventional RECIST so far, unequivocal LSR was observed in five patients (five lesions) using PERCIST criteria. For lesions with a largest tumor diameter of ≤3 cm (5/9), up until now, one LSR has been detected (29 mm). An example of a successfully treated lesion with a largest tumor diameter of ≤3 cm is shown in Fig. 1. Contrarily, all (4/9) lesions with a largest diameter of >3 cm recurred. CT-guided core biopsy confirmed tumor relapse in one patient who was successfully retreated with percutaneous IRE 4 months after the initial treatment; hereafter, no local recurrence was detected until his death (patient 1, Fig. 2) (patient died of brain metastases). Tumor recurrence distant from the pelvic treatment site developed in 4/9 patients: cerebral (*n* = 1), pulmonary (*n* = 2), and in a different site in the pelvis (*n* = 1). Lesion-based primary efficacy rate was 33% (3/9), and assisted efficacy rate was 44% (4/9). Adjusted for tumor size, the primary efficacy was 80 (4/5) and 0% (0/4) for tumors ≤3 and >3 cm, respectively. Assisted efficacy was 80 (4/5) and 25% (1/4), respectively. One day post-IRE, the reported pain was moderate with a median VAS score of 3 (range 0–5); pain could easily be controlled with acetaminophen combined with NSAIDs and opioids if needed. Prior to IRE, three patients reported debilitating pain; 3 months after IRE pain perception had remained unchanged in one patient (VAS score 5; patient 2) and had improved slightly (VAS score from 5 to 4; patient 5) and considerably (VAS score from 6 to 3; patient 3) in the other two patients.

## Discussion

Despite advances achieved in the radical treatment of primary colorectal and urogenital cancer, locoregional relapse remains a major therapeutic concern [1–4, 23]. This case series demonstrates the feasibility of IRE to achieve local tumor control (4/9 lesions, respectively, after one [*n* = 3] or two [*n* = 1] procedures) for heavily pre-treated pelvic tumor recurrences. Lesion size seems key in predicting outcome with only 1/4 LSR’s for lesions ≤3 cm. While safety should be confirmed in larger series, no major life-threatening complications occurred. Conversely, the number of patients suffering from permanent ablation-induced neural function loss should be considered high (6/8), which contradicts earlier animal studies [14, 16, 17, 24].

While tumor recurrences within the pelvis encompass a broad disease category, it is widely recognized that selected patients may benefit from intensive local therapy [25–27]. Surgical resection, often with curative intent, remains the treatment of first choice. However, the number of eligible patients is relatively small because the presence of adjacent crucial anatomical structures renders them unsuitable for complementary radical procedures [28]. Generally, the maximum tolerable radiation dose has already been reached during treatment of the primary tumor. In these cases, re-radiation is precluded due to the high risk of radiation-induced complications [4]. In the past two decades, thermal ablation has been investigated as treatment option for pelvic tumors. Apart from the (potentially) curative possibilities, thermal ablation can be valuable in the palliative setting to achieve cytoreduction and pain relief [9, 29]. For example, palliative CT-guided RFA for painful pelvic recurrences of rectal cancer is considered a feasible and effective treatment in selected cases where the recurrence is located at a safe distance from major nerves, intestines, and urogenital tract structures [29–32]. In one study, twelve patients who underwent RFA for pain reduction were pain-free at the end of follow-up [29]. With one rectovesical fistula (8%) and one rectal abscess (8%), complication rate was considered acceptable. Since all lesions in our series were deemed unsuitable for thermal ablation, a fair comparison to these reports cannot be made. Yet, either RFA as IRE can induce the occurrence of post-procedural fistulas via the needle tracks. Up till now, only two case reports have been published regarding the use of IRE for the treatment of pelvic malignancies. In both cases, no major complications occurred [33, 34].

The actual working mechanism of IRE is based on a nonthermal effect; nonetheless, the development of some heat seems inevitable. Van den Bos et al. [35] found a temperature rise of 19.6 °C at 5 mm distance from the electrodes (using 1500 V/cm) in a nonperfused gelatine tissue model. According to animal studies, transient nerve dysfunction starts at temperatures as low as 40 °C [36] and permanent nerve injury at 51 °C [37]. Since the conductivity and the electric field distribution may differ between healthy and tumor tissue, this may have resulted in focal areas with a greater temperature rise, resulting in permanent thermal damage to the nervous structures [38]. The discrepancy between the preclinical animal experiments and our results regarding neural damage remains largely unclarified. One possible explanation is that the interelectrode distance in the animal experiments was smaller [approximately 5 mm; 750 V (1500 V/cm)]. Given the stronger impact on temperature development of adjusting voltage versus adjusting interelectrode distance [35], the temperature rise will be higher for settings used in clinical practice [approximately 20 mm; 3000 V (1500 V/cm)]. Another explanation can be electric field heterogeneity, as this is key in determining IRE outcome [39]. Although the parameters used in the animal studies were largely in accordance with ours, the induced potential depends on the ohmic characteristics of the tissue, which can differ in inhomogeneous tissues [40]. In the future, sequential pulsing, with pulse trains of 20 or 30 pulses, may prove to reduce the maximum tissue temperature gradient and therefore the extent and volume of thermal damage [35]. However, further research is needed to assess whether this is factual and whether this does not compromise oncologic efficacy. Furthermore, as opposed to healthy sciatic nerves and surrounding tissues in the animal experiments, the neural and perineural structures in the presented patients may well have been injured to some extent by earlier treatments or by tumor compression; by undergoing the IRE procedure, they may have lost their neural function completely. Li et al. [14] explored the effect of IRE on nerves in a rat model and observed that nerve continuity was preserved post-procedurally. However, studies have shown that the regeneration process of nerves in larger animals is better comparable to neural regeneration in humans and might therefore be more suitable to study longer distance nerve regeneration [41, 42]. Schoellnast et al. [16, 17] assessed the acute, subacute, and delayed effects of IRE on the sciatic nerve in a pig model and concluded that IRE potentially damaged neural structures. They hypothesized that the observed preservation of the endoneurium architecture and the Schwann cell proliferation could potentially enable axonal regeneration. Most animals did not exhibit signs of lameness 1 month post-IRE. Nevertheless, nerve conduction studies revealed residual neural function loss in half of the animals. These inconsistent results may be explained by the fact that the gluteal, extensor, and adductor muscles of the thigh can partially compensate for sciatic nerve palsy. In contrast to healthy individuals, cancer patients are generally less physically active and have less capacity to use other muscles to compensate the loss of neural function. In addition, preexisting pain complaints might hinder optimal recovery post-IRE. Recently, Tam et al. [43] investigated the post-ablation effects of IRE in the epidural space of the porcine spine and concluded that even at low electric field strengths nerve root injury can occur (*p*  ≤ 0.05). To recognize impending nerve injury and to prevent irreversible damage, intraoperative neurophysiologic monitoring (IONM) might be helpful during percutaneous IRE, since neural structures adjacent to the target area are often poorly visible [44]. Chu et al. [45] confirmed that temporary axonal dysfunction can occur during IRE, and showed that IONM has the potential to detect conduction block at an early and reversible stage.

There were several inherent limitations in the present study. First, the study design was retrospective and the case number was limited. Second, the heterogeneity of tumor type and -size, anatomical location, and treatment indication (symptom palliation or disease control) was high. Another limitation was the inability to objectively quantify the level of nerve palsy after the ablation, especially given concomitant temporary muscle injury caused by IRE in some patients. To overcome this drawback in a future study, general neurological examination and nerve conduction studies could be performed to objectify the neural function pre- and post-IRE.

In conclusion, IRE may represent a suitable technique to treat well-selected locoregional tumors within the pelvis. However, as opposed to preclinical animal studies, permanent neural function loss can occur. Although complete ablation seems achievable for smaller lesions (<3 cm), this currently seems unrealistic for patients with larger tumors. For these patients, palliative care should be the primary focus.

## Abbreviations

ASA: American Society of Anesthesiologists
Ce: Contrast-enhanced
CT: Computed tomography
CTCAE: Common Terminology Criteria of Adverse Events
18F-FDG PET: 18F-fluorodeoxyglucose positron emission tomography
IONM: Intraoperative neurophysiologic monitoring
IRE: Irreversible electroporation
LSR: Local site recurrence
PNI: Peripheral nerve injury
RECIST: Response Evaluation Criteria in Solid Tumors
RFA: Radiofrequency ablation
SABR: Stereotactic ablative body radiation therapy
TDP: Time to distant progression
PERCIST: PET Response Criteria in Solid Tumors
TLP: Time to local progression
VAS: Visual Analog Scale
